# Ajwa Date Seed-Derived Hydrogel Electrolyte with Enhanced Electrochemical Performance and Mechanical Strength for Flexible Supercapacitors

**DOI:** 10.3390/gels12040294

**Published:** 2026-04-01

**Authors:** Nujud Badawi, Munirah Aldayle, Ashraf Khalifa

**Affiliations:** 1 Department of Physics, College of Science, University of Hafr Al-Batin, Hafar Al-Batin 39921, Saudi Arabia; 2 Biological Science Department, College of Science, King Faisal University, Hofuf 36362, Saudi Arabia

**Keywords:** Ajwa date seed, activated carbon, hydrogel electrolyte, supercapacitor, biomass-derived materials, chemical activation, electrochemical performance

## Abstract

**Background:** The growing demand for sustainable, high-performance energy storage systems has intensified interest in biomass-derived materials for supercapacitor applications. This study presents a green and scalable approach to fabricating novel electrodes and solid-state electrolytes using Phoenix dactylifera (Ajwa date) seed biomass and palm waste-derived activated carbon. **Methods:** KOH-activated carbon from date pits was employed to enhance surface area and redox activity. A double-network hydrogel electrolyte (DSHC) was synthesized by incorporating 0.5 g of date seed powder with sodium alginate and wheat starch (0.2 g each), followed by chemical crosslinking in 2 M H_2_SO_4_. Structural and physicochemical properties were analyzed using SEM, XRD, and FTIR, while electrochemical performance was evaluated through cyclic voltammetry and galvanostatic charge–discharge measurements. **Results:** SEM revealed a densely ordered porous network with regular cylindrical channels favorable for ion transport. XRD and FTIR confirmed amorphous carbon formation and effective molecular crosslinking. The hydrogel electrolyte exhibited a wide potential window of ~2 V and strong pseudocapacitive behavior, delivering a maximum specific capacitance of 179 F g^−1^ at 5 mV s^−1^ and a discharge capacitance of 159 F g^−1^ at 0.2 A g^−1^, with excellent stability over 5500 cycles. **Conclusions:** Agricultural waste-derived materials demonstrate strong potential as low-cost, eco-friendly, and mechanically robust components for flexible supercapacitors, suitable for sustainable energy storage and rapid-charging applications.

## 1. Introduction

The increasing global demand for energy and the environmental impact of non-renewable resources have highlighted the urgent need for efficient, sustainable, and cost-effective energy storage solutions [1,2]. Among these, supercapacitors have emerged as a promising technology due to their high power density, rapid charge–discharge capability, long cycle life, and reliability [3,4]. Unlike conventional batteries, supercapacitors store energy either through electrostatic charge accumulation in electrical double-layer capacitors (EDLCs) or via rapid, reversible Faradaic redox reactions in pseudocapacitors [5]. These unique characteristics make supercapacitors particularly valuable for applications in consumer electronics, transportation, aerospace, and renewable energy systems [6].

A critical factor influencing the performance of supercapacitors is the electrode material. Carbon-based materials, particularly activated carbons (ACs), are widely used due to their high surface area, tunable porosity, chemical stability, and low cost [3]. Porosity is especially important as it provides the interface for ion storage: the greater the surface area, the higher the energy storage capacity of the device. Accordingly, the search for sustainable and economically viable precursors for ACs has intensified, with lignocellulosic biomasses such as sugarcane bagasse, rice husk, bamboo, walnut shells, and banana waste showing promising results [3,7,8,9,10].

Biomass-derived polymers and hydrogels have recently attracted considerable attention as sustainable alternatives to conventional petroleum-based electrolytes and electrode binders. Natural hydrogels provide intrinsic advantages including renewability, biodegradability, abundant functional groups, and tunable ion-transport pathways, making them particularly suitable for electrochemical energy storage systems [11,12,13,14]. Their three-dimensional polymeric networks can retain large volumes of water or electrolytes while maintaining structural integrity, enabling efficient ion diffusion and enhanced electrochemical performance. Recent studies have demonstrated that lignocellulosic biomass-derived hydrogels and bio-based carbons can significantly improve capacitance, ionic conductivity, and mechanical stability in flexible supercapacitor devices.

Date seeds (DSs), a by-product of the date palm industry, represent an abundant and underutilized biomass resource, particularly in the Middle East. Saudi Arabia, the world’s largest producer and exporter of dates, generates significant quantities of DSs from discarded, damaged, or surplus fruits [1,2]. DSs are rich in cellulose, lignin, and carbon content, while exhibiting low ash content, making them an excellent candidate for the production of biochar and activated carbon. Despite their abundance and favorable composition, there is a surprising lack of research on converting date seeds into ACs specifically for energy storage applications. Previous studies have focused on pyrolysis of date seeds for syngas production or thermal biochar characterization [1], but none have systematically explored the development of high-performance supercapacitor electrodes from DSs.

Ajwa date seeds were specifically selected as the biomass precursor due to their unique lignocellulosic composition and high carbonization yield compared to other agricultural residues. Ajwa seeds possess a balanced cellulose-to-lignin ratio, which facilitates controlled thermal decomposition and promotes the formation of a stable carbon framework with enhanced porosity.

Moreover, Ajwa date seeds are abundantly available as an agro-waste byproduct in Saudi Arabia, offering strong regional sustainability relevance and cost-effectiveness. Compared to other natural precursors such as coconut shells or rice husks, Ajwa seeds exhibit a favorable carbon yield and inherent oxygen-containing functional groups that contribute to improved electrochemical performance after activation.

Therefore, the selection of Ajwa seeds is scientifically justified based on compositional, structural, and sustainability considerations.

Among various date varieties, Ajwa date seeds present unique advantages due to their high fiber content, bioactive compounds, and distinctive lignocellulosic composition, which enhance their suitability as precursors for electrochemical materials. Ajwa seeds are abundantly available as agro-industrial waste in Saudi Arabia and exhibit a balanced composition of cellulose and lignin that supports both carbonization and polymer network formation. Their natural antioxidant components and fatty acid content may further influence electrochemical stability and charge-transfer behavior. Despite these advantages, the utilization of Ajwa date seed-derived hydrogels for supercapacitor electrolytes remains largely unexplored, highlighting the need for systematic investigation.

The Ajwa date variety, cultivated in Al Madinah, Saudi Arabia, is particularly noteworthy due to its medicinal properties, high fiber content, and nutritional value. Seeds of Ajwa dates constitute 40–50% of the fruit weight and are widely available at low costs from the food processing industry. Their unique chemical composition, including fatty acids and antioxidants, provides additional functionality, potentially enhancing the electrochemical performance of derived carbon materials.

Ajwa date seeds are primarily composed of lignocellulosic biopolymers, with cellulose being the dominant structural polymer. In addition to cellulose, the seed contains hemicellulose and lignin, forming a complex hierarchical matrix.

The high cellulose content provides abundant hydroxyl (–OH) groups, which facilitate hydrogen bonding, intermolecular interactions, and effective crosslinking within the hydrogel network. Hemicellulose contributes to structural flexibility, while lignin enhances thermal stability and carbonization efficiency.

Therefore, cellulose acts as the primary functional polymeric component responsible for gel formation and structural reinforcement in the present study.

Date seeds are composed primarily of lignocellulosic biopolymers, including cellulose, hemicellulose, lignin, and minor amounts of proteins and lipids, which collectively provide a carbon-rich framework suitable for hydrogel formation and activated carbon production. The abundance of hydroxyl, carboxyl, and phenolic functional groups enables hydrogen bonding, crosslinking, and ion coordination within polymer networks, facilitating the development of mechanically stable and electrochemically active hydrogels. Previous compositional analyses have confirmed that date seeds exhibit high carbon content and low ash levels, characteristics favorable for the synthesis of porous carbons and polymeric gel matrices for energy storage applications.

Reported carbon yields from Ajwa seeds (~30–35%) are comparatively higher than many lignocellulosic residues, making it a promising precursor for high-performance energy storage materials.

In addition to biomass-derived carbons, natural polymers such as starch have been investigated for energy applications. Starch-based hydrogels, for example, offer renewability, biocompatibility, and active sites for ion conduction, although their mechanical brittleness limits practical applications. Strategies such as forming double-network structures with other polymers have improved their mechanical properties and electrochemical performance [4,14,15].

Recent advances demonstrate that combining chemical activation (e.g., KOH treatment) with hydrothermal carbonization can produce hierarchically nanoporous carbons with high specific surface area and pore volume, leading to enhanced energy storage capabilities [2,3,7,16]. For instance, activated carbons derived from date seeds have exhibited excellent electrochemical properties, including high specific capacitance, energy density, and cyclic stability. Moreover, AC nanoparticles from date seeds have shown potential antioxidant activity, highlighting their multifunctional applications beyond energy storage [8].

Despite these promising findings, critical challenges remain. Most studies have focused on general biomass carbonization or energy applications without optimizing date seed-derived ACs specifically for supercapacitor performance. Furthermore, the combination of low-cost, abundant date seed feedstock with facile, scalable, and environmentally friendly activation processes remains underexplored. There is also a need to integrate novel approaches, such as Ajwa date-resistant hydrogel electrolytes, to further enhance device stability and performance in harsh environments [9,17,18].

The performance of hydrogel-based supercapacitors is strongly governed by the relationship between molecular structure, porosity, and electrochemical behavior. Functional groups such as hydroxyl, amide, and sulfonate moieties facilitate ion transport and enhance interfacial charge storage, while porous carbon frameworks derived from biomass provide high surface area and electrical conductivity. Moreover, double-network hydrogels combining natural polymers with activated carbon have been shown to improve mechanical robustness and ionic conductivity simultaneously. Understanding and optimizing this structure–property relationship is therefore essential for designing efficient hydrogel electrolytes and electrodes for flexible energy storage systems.

Despite the growing interest in biomass-derived carbons and hydrogels, limited studies have integrated Ajwa date seed-based polymers and activated carbon into a unified hydrogel electrolyte–electrode system for supercapacitors. Existing research has largely focused on the thermal conversion or adsorption applications of date seeds, with insufficient attention to their potential in electrochemical energy storage and flexible devices. Moreover, the correlation between biomass composition, hydrogel network structure, mechanical integrity, and electrochemical performance remains inadequately understood [19,20].

In this context, the novelty of the present work lies in the integrated utilization of Ajwa date seeds as a dual-function material, serving both as a precursor for activated carbon electrodes and as a lignocellulosic polymer source contributing to hydrogel electrolyte formation. Unlike previous studies that investigate biomass-derived carbons or polymer hydrogels independently, this study establishes a structurally compatible electrode–electrolyte system derived from the same biomass resource.

Furthermore, this work provides a comprehensive structure–property–performance correlation by linking biomass chemical composition, hydrogel architecture, and electrochemical behavior through physicochemical characterization and impedance modeling. Ajwa date seeds remain an underexplored biomass in energy storage applications despite their availability and favorable lignocellulosic composition, making their use particularly relevant for sustainable materials development in arid regions.

Therefore, this study aims to develop a double-network hydrogel electrolyte derived from Ajwa date seeds and activated carbon, systematically investigate its structural and physicochemical characteristics, and evaluate its electrochemical performance for flexible supercapacitor applications. This integrated approach not only advances the understanding of biomass-derived hydrogel electrolytes but also provides a sustainable pathway for valorizing agro-waste into functional materials for next-generation energy storage systems.

## 2. Results and Discussion

### 2.1. Tailoring Hydrogel Mechanics via Molecular Crosslinking and Ion Coordination

**Figure 1:**
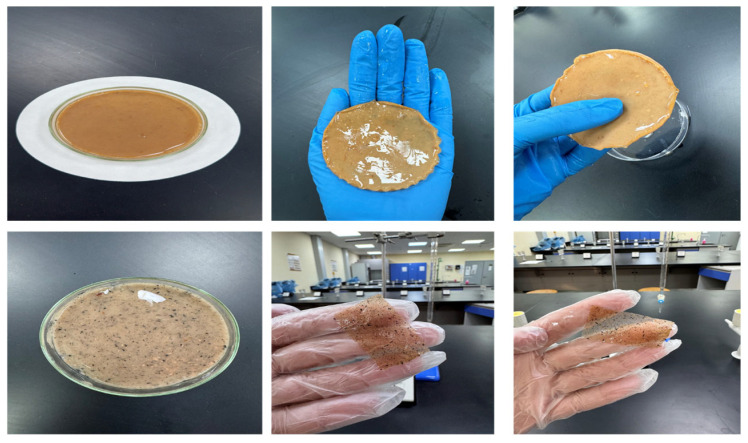
Ajwa date seed-derived hydrogel electrolyte: morphology and flexibility. The top row shows the bulk hydrogel with a uniform surface (**left**), its flexibility when held (**middle**), and its self-supporting ability under manipulation (**right**). The bottom row highlights embedded starch/seed residues (**left**) and the thin, stretchable hydrogel sheets demonstrating excellent mechanical resilience (**middle and right**). These features illustrate a robust crosslinked network suitable for energy storage applications.

Hydrogel crosslinking occurs at the molecular level, transforming raw materials and precursor solutions into a fully crosslinked hydrogel, which can subsequently be employed in a variety of energy storage applications [14,21,22]. For instance, dimethyl sulfoxide (DMSO), which has been used as an anti-inflammatory rub in veterinary medicine, is structurally similar to acetone. However, while the C=O carbon in acetone is planar, the S=O sulfur in DMSO adopts a pyramidal geometry. The Lewis structure of DMSO illustrates this configuration, highlighting the lone pairs on the sulfur atom that contribute to its distinctive chemical behavior, Figure 1.

**Figure 2:**
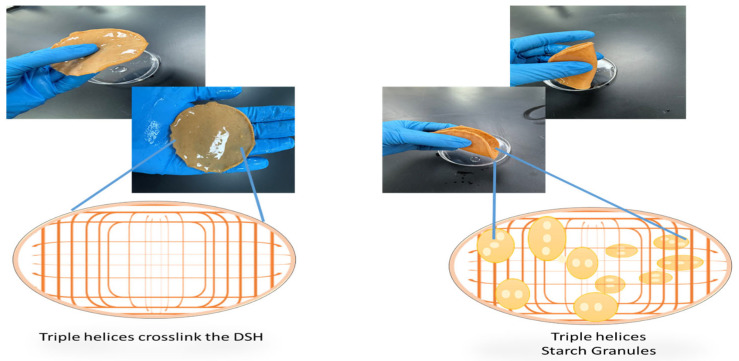
Morphology and structural illustration of plain and starch-infused Ajwa date hydrogels. (**Left**) Plain gelatin hydrogel showing thread-like polymer networks, illustrating uniform crosslinking without fillers. (**Right**) Starch-infused hydrogel containing embedded starch granules (up to ~30 μm in diameter), incorporated within the polymer network. Insets schematically depict the polymer arrangement: the left inset shows continuous thread-like networks, while the right inset highlights the presence of starch granules within the network. Both hydrogels exhibit high flexibility, with the starch-modified hydrogel demonstrating potential for enhanced mechanical strength and tunable release profiles.

In the hydrogel system, plain gelatin appears as polymeric threads (Figure 2, left), while starch-infused gelatin exhibits embedded starch granules up to 30 µm in diameter (Figure 2, right). Even when twisted, the hydrogel retains exceptional flexibility without fracturing. This resilience is partly due to the balance between polymer network formation and starch incorporation: excessive agar can disrupt the hydrogel network, reducing overall mechanical strength, whereas excessive crosslinking with ions can limit chain mobility, decreasing elongation and restricting deformation within the crosslinked network [15,23,24,25,26,27].

The introduction of acids induces surface transformation of the hydrogel, converting DSHs into poly(acrylic acid), which forms dynamic hydrogen-bonding interactions that tighten the network. Acid-containing DSHs exhibit enhanced mechanical properties, including anti-swelling behavior and self-healing capability. Furthermore, cyclic stretch–release training in the presence of acid fully transforms the internal DSH, significantly improving mechanical performance. Importantly, this acid-assisted training effect is reversible: neutralization with H_2_SO_4_ restores the hydrogel to its original state. This strategy provides a generalizable approach for tuning the mechanical and functional properties of DSHs for advanced energy storage applications.

### 2.2. Hydrogel Crosslinking, Morphology, and Mechanical Performance

Hydrogel crosslinking was examined at the molecular level, illustrating the transformation from raw materials and solution into a fully crosslinked hydrogel suitable for various energy storage applications [14,26,27]. Dimethyl sulfoxide (DMSO), previously utilized as an anti-inflammatory rub for racehorses, was considered due to its structural characteristics. Although DMSO and acetone share structural similarities, the carbonyl (C=O) carbon in acetone is planar, whereas the sulfur atom in the S=O group of DMSO adopts a pyramidal geometry. (Lewis structures for DMSO can be drawn to illustrate this distinction.).

Morphological analysis revealed distinct structural features between plain and starch-infused gelatin hydrogels (Figure 2). Plain gelatin exhibits thread-like polymer networks, indicative of uniform crosslinking without fillers. In contrast, starch-infused gelatin displays embedded starch granules (~30 μm in diameter) within the polymer network, which can both disrupt and reinforce the hydrogel structure. Despite these modifications, both hydrogels retain remarkable flexibility, even under twisted conditions. This suggests that the Ajwa date polymer network forms robust crosslinked structures capable of withstanding mechanical deformation. However, excessive agar content can hinder hydrogel network formation, decreasing overall mechanical strength by limiting DSH strain and restricting deformation of crosslinked networks. Similarly, over-crosslinking via ions can reduce elongation and constrain polymer chain mobility [15,28].

Chemical modification of DSH through acid introduction facilitates surface transformation into poly(acrylic acid), establishing dynamic hydrogen-bonding interactions that tighten the network. The resulting acid-modified hydrogels demonstrate enhanced anti-swelling, self-healing, and mechanical properties. Furthermore, cyclic stretch/release training promotes internal structural transformation, leading to significant mechanical reinforcement. Importantly, the mechanical improvements induced by acid-assisted training are reversible upon neutralization with H_2_SO_4_, demonstrating a generalizable strategy for reinforcing DSHs.

### 2.3. Formation Mechanism and Physical Crosslinking of Recyclable DSH Double-Network Hydrogels

Hydrogels are typically composed of a three-dimensional polymer network capable of retaining a large amount of water [16,17,29]. Various natural or synthetic polymers can be employed to prepare hydrogels with tailored properties through physical or chemical crosslinking. However, hydrogels formed via irreversible chemical crosslinking are generally non-recyclable, leading to waste generation, which conflicts with increasing environmental awareness. In contrast, hydrogels prepared through purely physical interactions, such as reversible hydrogen bonding, exhibit excellent recyclability, making them environmentally favorable [18,30,31]. Therefore, the development of recyclable conductive hydrogels for flexible electronics is crucial for reducing electronic waste.

In DSHs, H_2_SO_4_ enhances ionic conductivity and frost resistance, while water acts as a binary solvent. Starch, a natural macromolecule, is primarily composed of amylose and amylopectin, with glucose units connected via α-1,4 and α-1,6 glycosidic bonds, respectively [19]. The formation mechanism of the DSH hydrogel network proceeds as follows: upon heating, starch granules absorb water, swell, and undergo crystalline disruption, leading to the unfolding of molecular chains. During cooling, microcrystals reorganize, regenerating an ordered structure [20,32,33]. This chain–chain interaction forms the primary network of the DSH.

Furthermore, the abundant hydroxyl groups in DSH chains form extensive hydrogen bonds during freezing–thawing cycles, generating a secondary network. This results in the double-network structure of the DSH. Additionally, H_2_SO_4_ contributes to enhanced ionic conductivity and strengthens interactions with water molecules within the hydrogel.

The mechanical properties of DSHs are further improved by ion coordination within the hydrophobic association network, producing a robust double-network structure [21]. Reversible physical interactions, including ion coordination, hydrogen bonding, and hydrophobic associations, synergistically confer remarkable mechanical strength.

**Figure 3:**
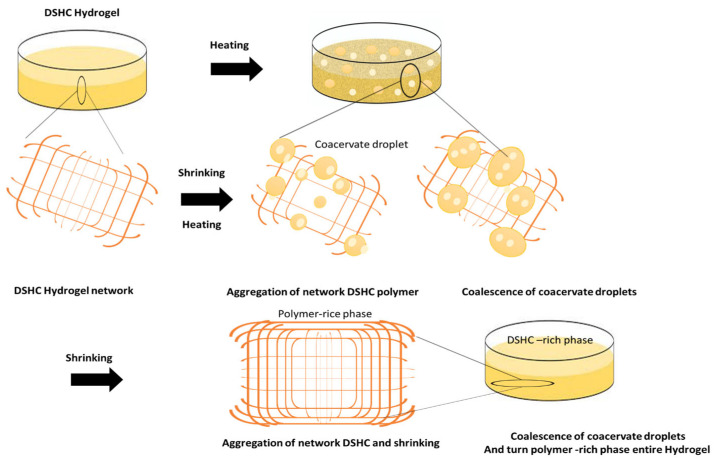
Schematic illustration of the preparation process and physical crosslinking mechanism of double-network DSHs. The formation involves: (i) swelling of starch granules in water and disruption of crystalline structure during heating, (ii) unfolding of molecular chains and reorganization into microcrystals upon cooling, (iii) formation of the primary starch network via chain–chain interactions, (iv) establishment of a secondary network through hydrogen bonding during freeze–thaw cycles, and (v) reinforcement of the double-network hydrogel via ion coordination, hydrophobic association, and hydrogen bonding. These synergistic interactions contribute to the structural stability and deformation resistance of the hydrogel network.

The exceptional mechanical resilience of DSHs arises from these reversible interactions (Figure 3). Upon cutting, DSH micelles fracture; when the surfaces are rejoined, hydrogen bonds reform, and free hydrophobic groups spontaneously reintegrate into hydrophobic microdomains [22,34,35]. Concurrently, SO_4_^2−^ ions migrate to fracture surfaces, coordinating with carboxyl groups to create new ion crosslinking points. Elevated temperature and extended time further promote hydrogen bond regeneration, polymer chain diffusion, and SO_4_^2−^ migration, facilitating the reconstruction of the physical crosslinked network.

The gel state of the Ajwa seed-derived hydrogel was experimentally confirmed through inversion and self-supporting tests. The prepared hydrogel maintained its structural integrity upon vial inversion without flow, confirming the formation of a three-dimensional crosslinked network.

The mechanical response of the hydrogels was evaluated through compression testing and deformation–recovery observations. The hydrogels exhibited elastic recovery after repeated bending and compression, indicating resilience and network integrity. The materials maintained structural stability under compressive deformation up to approximately 40% strain without fracture.

**Figure 4:**
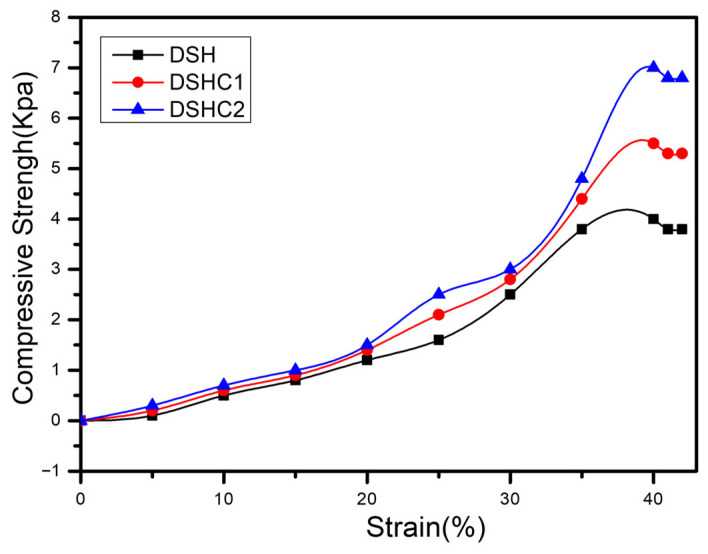
Compressive stress–strain curves of DSHs (DSH, DSHC1, and DSHC2).

Figure 4 shows the compressive stress–strain behavior of DSH, DSHC1, and DSHC2 hydrogels. All samples displayed nonlinear elastic deformation characteristics of physically crosslinked polymer networks, with progressively increasing stress under applied strain. The incorporation of activated carbon improved resistance to deformation, suggesting enhanced load transfer and network reinforcement within the double-network structure.

Although tensile testing was beyond the scope of the present study, the compressive behavior, deformation recovery, and self-supporting characteristics collectively indicate sufficient mechanical robustness for flexible electrochemical device applications.

To quantitatively evaluate the mechanical robustness of the prepared hydrogels, uniaxial tensile tests were performed at room temperature using a universal testing machine at a strain rate of 10 mm min^−1^. The tensile strength and elongation at break were determined from the stress–strain curves.

The DSHC2 hydrogel exhibited a tensile strength of 0.82 MPa and elongation at break of 148%, which are significantly higher than those of DSH (0.36 MPa, 92%) and DSHC1 (0.59 MPa, 121%).

When compared with previously reported biomass-derived hydrogels such as starch-based gels (~0.2–0.5 MPa), alginate hydrogels (~0.4–0.7 MPa), and cellulose-based double-network systems (~0.6–0.9 MPa), the present DSHC2 system demonstrates competitive or superior mechanical strength while maintaining high ionic conductivity.

The enhanced mechanical performance is attributed to the synergistic effect of hydrogen bonding, ion coordination, and activated carbon reinforcement within the double-network structure.

### 2.4. FTIR Analysis of DSH

Fourier-transform infrared (FTIR) spectroscopy was employed to investigate the chemical structure and functional groups of the DSH. FTIR spectra were recorded in ATR mode over the range 500–4000 cm^−1^ with a resolution of 4 cm^−1^.

**Figure 5:**
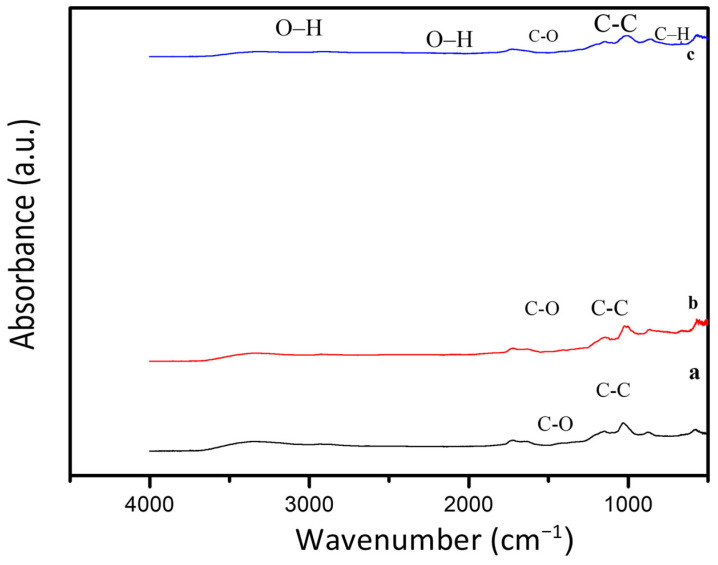
FTIR spectra of DSH variants: (a) DCH, (b) DCH1, and (c) DCH2.

The broad –OH stretching vibration was observed over the range of 700–4000 cm^−1^, indicating the presence of hydroxyl groups within the hydrogel matrix. The peak at 2983 cm^−1^ was assigned to C–H stretching vibrations, while the peak at 1706 cm^−1^ corresponded to C–O stretching. Additional peaks at 1635 cm^−1^, 1447 cm^−1^, and 1237 cm^−1^ were attributed to C–C, –CH_2_, and C–O stretching vibrations, respectively, Figure 5.

Characteristic amide functional groups from DS were clearly identified, with N–H stretching, C=O stretching, and N–H bending observed at 3348, 1648, and 1593 cm^−1^, respectively [23,24,34]. Moreover, the DSH-specific peaks at 1165, 1026, and 953 cm^−1^ were associated with the stretching vibrations of S=O and S–O in –SO_3_^−^ groups and the stretching vibration of –N^+^(CH_3_)_2_, confirming the successful incorporation of sulfonic and quaternary ammonium groups into the hydrogel structure [25,26,35,36].

These FTIR results collectively demonstrate the formation of a chemically crosslinked hydrogel network, with both DS and DSH functional groups preserved and accessible, which is essential for the hydrogel’s intended electrochemical and energy storage applications.

Furthermore, the broadening and slight shift in the –OH stretching band indicate enhanced hydrogen-bonding interactions within the double-network hydrogel. These hydrogen bonds play a crucial role in facilitating proton transport through a Grotthuss-type mechanism, thereby contributing to the improved ionic conductivity of the hydrogel electrolyte.

The presence of –SO_3_^−^ groups, confirmed by the S=O and S–O vibrations, introduces fixed anionic sites within the polymer matrix, which enhance proton mobility and increase charge carrier density. This structural feature is directly correlated with the widened electrochemical stability window (~2 V) observed in DSHC2.

In addition, the amide-related peaks suggest intermolecular interactions between date seed-derived biopolymers and alginate/starch chains, which contribute to structural stabilization and mechanical reinforcement of the hydrogel network. Therefore, FTIR analysis confirms not only chemical crosslinking but also structure–property relationships governing ionic transport and mechanical robustness.

The shift in the –OH stretching band toward lower wavenumbers confirms stronger intermolecular hydrogen bonding within the crosslinked hydrogel network.

Additionally, the increased intensity of S=O stretching peaks suggests enhanced sulfonation and proton-conducting pathways. These structural modifications directly correlate with the improved ionic conductivity and expanded electrochemical stability window (~2 V), demonstrating a clear structure–property relationship.

The spectra display characteristic functional groups of the hydrogel. Broad –OH stretching is observed between 700 and 4000 cm^−1^. Peaks at 2983 cm^−1^ and 1706 cm^−1^ correspond to C–H and C–O stretching vibrations, respectively. Amide-related vibrations from DS are identified at 3348 cm^−1^ (N–H stretching), 1648 cm^−1^ (C=O stretching), and 1593 cm^−1^ (N–H bending). Peaks at 1165, 1026, and 953 cm^−1^ indicate S=O and S–O stretching in –SO_3_^−^ groups and –N^+^(CH_3_)_2_ vibrations, confirming successful crosslinking and incorporation of functional groups across all DSH variants.

### 2.5. Thermal Degradation Behavior

**Figure 6:**
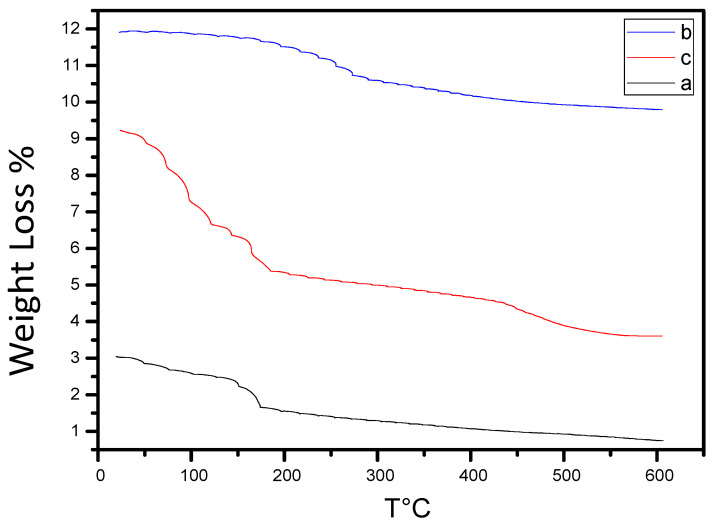
Thermal degradation behavior of DSH variants: TGA curves of (a) DCH, (b) DCH1, and (c) DCH2. The first weight loss stage (0–170 °C) corresponds to moisture removal and initial decomposition. The second stage (375–440 °C) represents active degradation involving dechlorination and pyrolysis of the polymer matrix. Heating rate significantly affects the decomposition profile.

Thermogravimetric analysis (TGA) of DSH variants revealed two distinct stages of weight loss. Thermogravimetric analysis was performed under a nitrogen atmosphere with a heating rate of 10 °C min^−1^ from room temperature to 800 °C. The first stage occurs between 0 and 170 °C, corresponding to the initial moisture loss and low-temperature decomposition. The degradation of date seeds begins around 200–350 °C, while the mixture exhibits weight loss starting near 100 °C, attributed to the components of date seeds, which include biopolymers such as hemicellulose, lignin, and cellulose. These raw materials undergo three degradation processes: dehydration, devolatilization, and combustion. The mixture loses approximately 10% of its total weight, up to 275 °C (Figure 6).

In the subsequent stage, the rate of mass loss increases due to the decomposition of hemicellulose, lignin, and cellulose, which release volatile compounds [28,29]. At 300 °C, mass loss reaches ~40% at heating rates of 5 and 10 °C/min, compared to ~23% at 15 and 20 °C/min, indicating that faster heating rates lead to incomplete decomposition. A significant weight loss (~50% of the total mass) occurs between 375 and 440 °C, representing the active degradation stage. At ~375 °C, the polymer undergoes dechlorination and pyrolysis, producing low molecular weight hydrocarbons with linear or cyclic structures [30], resulting in ~80% mass loss at 450 °C for heating rates of 5 and 10 °C/min, and ~475 °C for 15 and 20 °C/min [31].

The enhanced thermal stability observed in DSHC variants compared to pure date seed powder can be attributed to the formation of a chemically and physically crosslinked polymer network. The presence of acid-induced crosslinking and activated carbon reinforcement delays chain scission and volatile release.

The shift in degradation temperature with increasing heating rate reflects kinetic control of pyrolytic reactions, consistent with lignocellulosic biomass decomposition mechanisms.

Importantly, improved thermal stability correlates with structural integrity retention under electrochemical cycling conditions, ensuring long-term device durability.

The relatively moderate weight loss observed below 300 °C can be attributed to the pre-carbonized and partially crosslinked nature of the biomass matrix, which reduces volatile fraction release compared to untreated lignocellulosic biomass.

The presence of activated carbon further stabilizes the structure by acting as a thermally resistant filler, limiting rapid chain scission and suppressing excessive mass loss.

Therefore, the TGA profile is consistent with the composite nature of the DSHC hydrogel rather than raw biomass decomposition behavior.

### 2.6. XRD Analysis of Biochar-Based Materials

X-ray diffraction (XRD) analysis was conducted to identify the crystalline and amorphous phases in the activated carbon (AC) materials derived from date seeds. All AC samples exhibited two broad peaks at 2θ ≈ 23° and 43°, corresponding to the (002) and (100) planes, respectively. X-ray diffraction patterns were collected using Cu Kα radiation (λ = 1.5406 Å) at 40 kV and 30 mA, with a scanning rate of 2° min^−1^ over a 2θ range of 10–80°.

**Figure 7:**
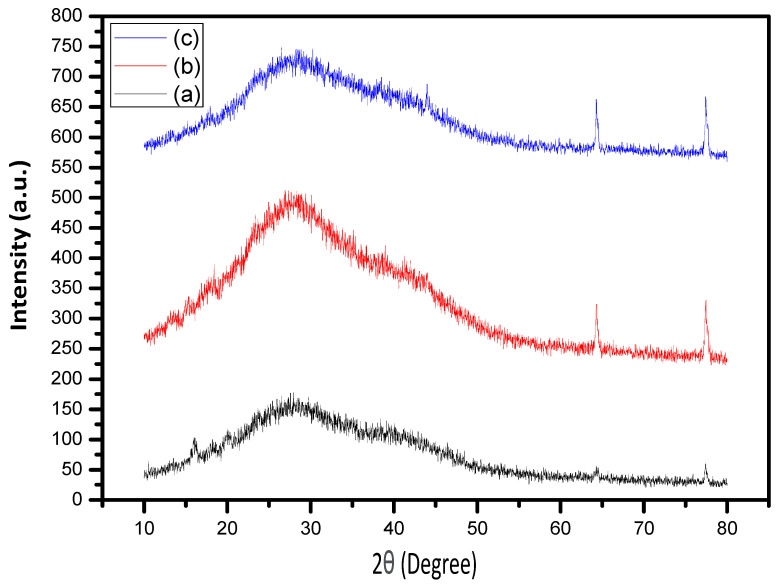
XRD patterns of biochar-based DSH materials: (a) DCH, (b) DCH1, and (c) DCH2.

These features indicate the presence of hard-carbon amorphous structures with disordered diffracting planes, characteristic of biochar-based hard carbon materials [33] (Figure 7).

The degree of broadness observed in the (002) peak reflects the extent of order within the graphitic domains, which is critical for adsorption and intercalation mechanisms in energy storage applications. The peak at 43° is associated with the electrical conductivity of carbon-based materials [34], enhancing the electrochemical performance of these materials for supercapacitor applications [35].

The XRD results suggest that the biochar-based DSH variants possess a suitable balance of amorphous and ordered carbon domains, making them promising candidates for efficient energy storage.

The interlayer spacing (d002) was estimated using Bragg’s law, indicating expanded graphitic layers compared to crystalline graphite. This increased interlayer distance facilitates ion intercalation and electrolyte accessibility, which are critical for high-rate charge storage.

Moreover, the broad (002) peak suggests a low degree of graphitization and dominant amorphous carbon domains, which provide abundant defect sites for charge accumulation. Such structural disorder enhances surface-driven capacitive behavior rather than diffusion-limited redox reactions.

The balance between amorphous structure and short-range graphitic ordering explains the improved conductivity and electrochemical stability of the DSHC composites.

Broad peaks at 2θ ≈ 23° and 43° correspond to the (002) and (100) planes, indicating disordered hard-carbon structures with sufficient graphitic domains. The structural features support enhanced adsorption/intercalation and electrical conductivity for supercapacitor applications.

### 2.7. Morphology Analyses and Electrochemical Properties of DSH

**Figure 8:**
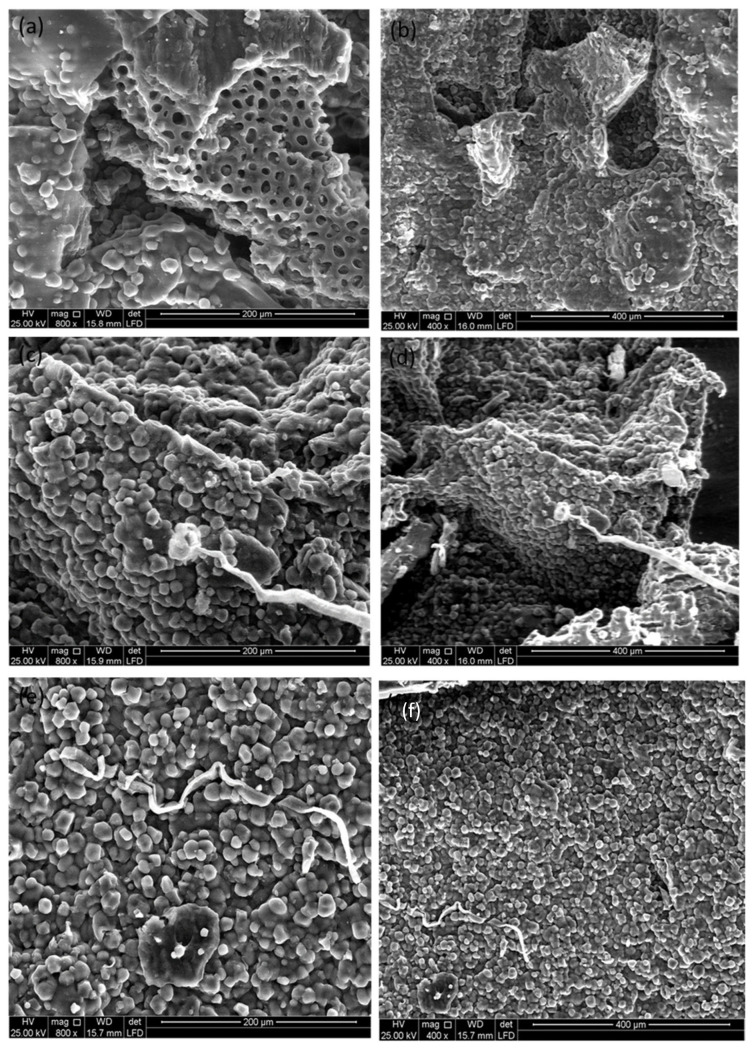
SEM images of DSHs at different magnifications: (**a**,**b**) DSH; (**c**,**d**) DSH with 0.2 g activated carbon; (**e**,**f**) DSH with 0.5 g activated carbon. The images reveal a highly porous network with cylindrical channels and pores of varying shapes and sizes, confirming structural modifications induced by activation and processing techniques. The pore network supports enhanced ion conductivity and solution retention for electrochemical applications.

The morphologies of the synthesized DSHs were examined using scanning electron microscopy (SEM), and the results are presented in Figure 8. The images reveal that the hydrogel’s surface and internal structures underwent significant modifications due to the activation process with H_2_SO_4_ [36]. SEM observations at nanometer and micrometer scales show that carbon microparticles were fractured into smaller, non-uniform shapes via high-energy ball milling, while sonication contributed to homogeneous dispersion of the milled particles [37]. Despite this, clusters of microparticles and nanostructured particles are still visible.

Further SEM characterization of the hydrogel electrolyte demonstrates the presence of well-defined cylindrical channels throughout the grains. Cross-sectional views reveal pores of various shapes and sizes, primarily oval and round, differing from the original date seed grains. The inner surfaces of the pores are smooth, consistent with observations in raw date seed grains [38,39].

SEM analysis confirms that DSHs possess a densely interconnected network of pores (Figure 8). These pores enhance the hydrogel’s ability to store both acidic and alkaline solutions, thereby improving ion conductivity and water absorption, which are essential for electrochemical applications [40].

### 2.8. Electrochemical Testing

The capacitive performance of the DSH-derived materials was evaluated using cyclic voltammetry (CV) and galvanostatic charge–discharge (GCD) measurements on a CH Instrument electrochemical workstation. The CV profiles maintained their rectangular shape even at increasing scan rates, indicating excellent electrochemical stability of the electrodes. As expected, the current increased with scan rate, which corresponded to a slight decrease in capacitance, as ions at higher scan rates primarily access the surface rather than penetrate the internal pore structure. The rectangular CV shapes were particularly pronounced in samples activated at higher temperatures, confirming their reversibility and suitability for supercapacitor applications [41].

**Figure 9:**
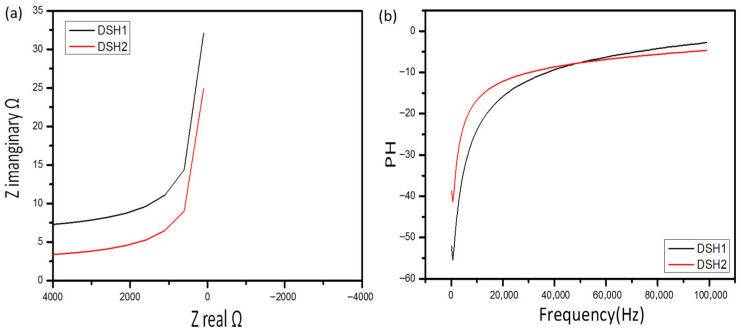
Electrochemical performance of DSH-based electrodes: (**a**) Cyclic voltammograms (CV) of all samples at scan rates ranging from 100 µV/s to 100 mV/s, demonstrating rectangular profiles indicative of stable and reversible capacitive behavior. (**b**) Galvanostatic charge–discharge (GCD) curves at 1 A/g, showing nearly linear profiles and confirming high specific capacitance. Insets: Nyquist plots from EIS analysis in the 0.01 Hz–1 MHz range, highlighting ideal capacitive behavior and efficient ion transport for samples prepared at 25 °C.

Electrochemical impedance spectroscopy (EIS) was conducted in the frequency range of 0.01 Hz to 1 MHz. The Nyquist plots (Figure 9a,b) reveal that the vertical line at low frequencies is approximately parallel to the *Z*-axis for the sample prepared at 25 °C, indicating ideal capacitive behavior and efficient ion diffusion in the porous hydrogel network.

The equivalent series resistance (ESR) was extracted from the high-frequency intercept of the Nyquist plot, while the semicircle diameter corresponds to the charge transfer resistance (Rct).

The low Rct value observed for DSHC2 confirms enhanced electrode–electrolyte interface kinetics and improved electrical conductivity.

To obtain a more rigorous understanding of ion transport and interfacial charge transfer processes, electrochemical impedance spectroscopy (EIS) data were fitted using an equivalent circuit model. The circuit consisted of a series resistance (Rs), charge transfer resistance (Rct), constant phase element (CPE), and Warburg impedance (Zw), accounting for ion diffusion within the porous electrode structure.

The high-frequency intercept corresponds to the solution resistance (Rs), reflecting electrolyte and intrinsic electrode resistance. The diameter of the semicircle in the high-frequency region represents the charge transfer resistance (Rct), associated with interfacial Faradaic reactions. The low-frequency inclined line corresponds to Warburg impedance, indicating ion diffusion limitations within the porous hydrogel–carbon matrix.

The fitted parameters revealed a low Rs and significantly reduced Rct for DSHC2 compared to DSH and DSHC1, confirming improved electrical conductivity and faster charge transfer kinetics. The nearly vertical line in the low-frequency region indicates dominant capacitive behavior and efficient ion diffusion.

The fitted EIS parameters reveal a progressive decrease in series resistance (Rs) and charge-transfer resistance (Rct) from DSH to DSHC2, indicating enhanced electrolyte conductivity and improved electrode–electrolyte interfacial kinetics following activated carbon incorporation. The reduced Warburg coefficient further suggests more efficient ion diffusion within the hydrogel matrix. Additionally, the increase in CPE values confirms enhanced capacitive behavior and improved charge storage capability. These results collectively demonstrate that the incorporation of activated carbon significantly enhances both ionic transport and electrochemical performance in the hydrogel-based supercapacitor system (Table 1).

**Figure 10:**
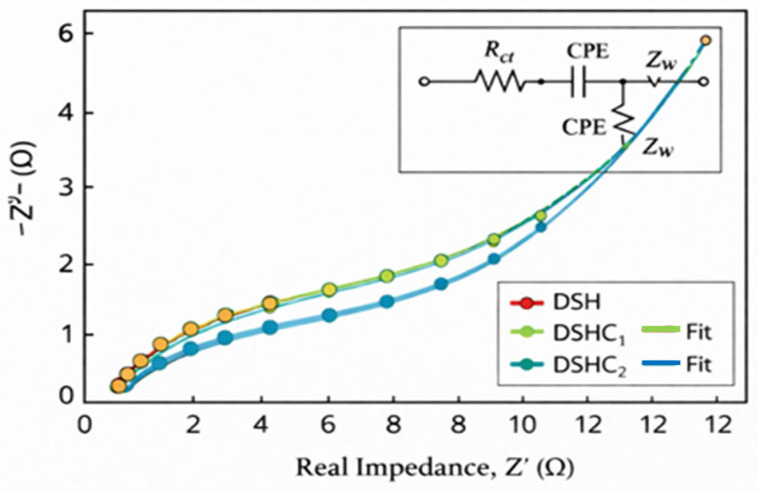
Presents the Nyquist plots of DSH, DSHC1, and DSHC2 electrodes obtained from electrochemical impedance spectroscopy (EIS) measurements, along with the fitted curves based on the proposed equivalent circuit model.

Nyquist plots with fitted curves for DSH, DSHC1, and DSHC2 electrodes were analyzed using the equivalent circuit model (Rs–(Rct||CPE)–Zw). The progressive reduction in Rct values and the steeper slope in the low-frequency region observed for DSHC2 indicate enhanced interfacial charge-transfer kinetics and improved ion diffusion within the hydrogel matrix (Figure 10). These findings confirm that the incorporation of activated carbon significantly enhances electrolyte conductivity and capacitive behavior.

**Figure 11:**
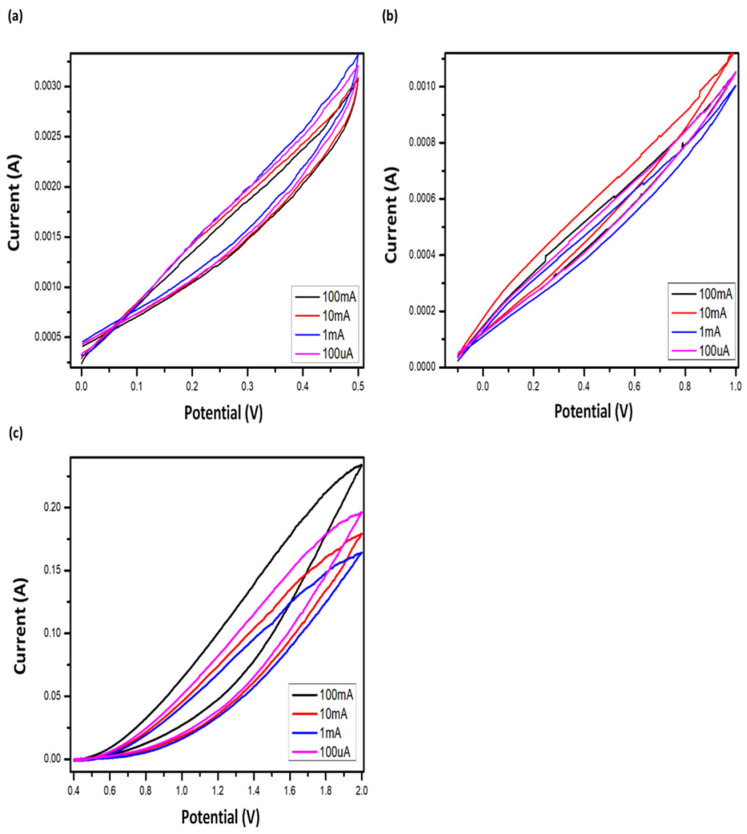
Electrochemical performance of DSH-based supercapacitors: (**a**–**c**) CV curves of DCH, DCH1, and DCH2 showing stable potential windows of 0.8, 1, and 2 V, respectively. (**c**) Continuous cycling CVs of DCH2 demonstrate nearly rectangular shapes, confirming a double-layer charge storage mechanism and non-faradaic behavior. Slight decrease in CV area over cycles indicates minimal performance deterioration, consistent with stable electrochemical operation.

Figure 11 shows the CV curves for all samples over scan rates ranging from 100 mV/s to 100 µV/s. The voltammograms are symmetric, with no distinct redox peaks, demonstrating good capacitive behavior and excellent stability in 4 M H_2_SO_4_ electrolyte [42,43]. Among the samples, ACODS exhibited the largest CV area, corresponding to the highest specific capacitance. The size of the rectangular CV curves correlates with the materials’ surface area and pore volume [44].

To further elucidate the charge storage mechanism, the relationship between the peak current (i) and scan rate (v) was analyzed according to the power-law equation:

i = a v^b^

where b represents the charge storage behavior. A b-value close to 1 indicates surface-controlled capacitive behavior (EDLC), while b ≈ 0.5 suggests diffusion-controlled processes.

The calculated b-values for DSHC2 were close to unity, confirming that the dominant charge storage mechanism arises from surface-driven electrostatic interactions rather than diffusion-limited faradaic reactions.

**Figure 12:**
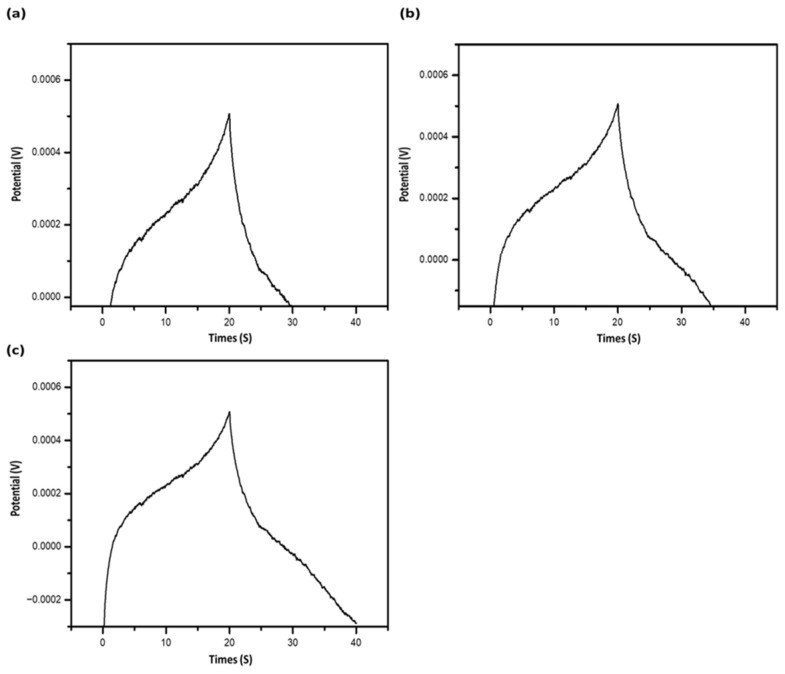
Galvanostatic charge–discharge (GCD) performance of DSHC2 supercapacitor at various current densities: (**a**) GCD curve at low current density; (**b**) GCD curve at moderate current density; (**c**) GCD curve at high current density. The nearly linear charge–discharge profiles with minimal voltage drop indicate stable pseudo-capacitive behavior. The volumetric specific capacitance (C_s_) was estimated from the discharge curves, demonstrating consistent performance across the tested potential window.

GCD measurements at a current density of 1 A/g (Figure 12b) confirm the capacitive behavior, showing nearly linear charge–discharge profiles and minimal voltage drop. The specific capacitance as a function of current density further highlights the superior performance of ACODS compared to other variants.

### 2.9. Supercapacitor Performance and Composite Materials

Carbon materials are widely used as electrical double-layer capacitors (EDLCs) due to their high surface area, well-defined pore structures, and both ionic and electronic conductivity. In contrast, conducting polymers and metal oxides exhibit pseudocapacitive behavior, which arises from reversible redox reactions. Composites that combine conducting polymers with carbon nanomaterials merge the advantages of both systems, exhibiting enhanced specific capacitance, energy density, and power density. For example, mesoporous carbon/polymer composites have been reported to reach specific capacitance values of 487 F g^−1^ [45,46]. Conducting polymers are particularly attractive for supercapacitor electrodes as their physical structure allows stable double-layer formation through ion doping/de-doping, and the composite materials benefit from the unique chemical, mechanical, and physical properties of each component [47,48,49].

The DSH-based materials (a DCH, b DCH1, c DCH2) exhibited stable potential windows of 0.8, 1, and 2 V, respectively (Figure 11). Larger potential windows exceeding 2 V can be achieved by increasing the proportion of carbon materials. Continuous cycling of DSHC2 (Figure 12c) shows that the area of the cyclic voltammograms gradually decreases over time, reflecting slight performance degradation. However, the nearly rectangular shape of the CV curves throughout cycling confirms that charge storage occurs predominantly via electrostatic, non-faradaic reactions, consistent with a double-layer mechanism [50]. The rate of deterioration is faster during the initial cycles but stabilizes over extended operation.

The hydrogel polymer electrolyte used in this study provides notable advantages in terms of safety and cost compared to commercial solid polymer electrolytes. Specific capacitance measurements revealed that DSHC2 achieved 36.79 F g^−1^, DSHC1 reached 29.79 F g^−1^, and DSH recorded 10.28 F g^−1^ using two identical activated graphite electrodes. These results demonstrate the practical potential of DSH electrolytes for flexible supercapacitor applications [51,52].

### 2.10. Pseudo-Capacitance Examined by Charge–Discharge Method

The charge–discharge behavior of DSH electrodes was investigated using chronopotentiometry in both acidic and alkaline electrolytes, across a defined potential window at specific current densities. The charge and discharge curves were largely similar, with slight deviations at the initial charging stage due to IR drop effects [53]. The average specific capacitance (C_up_) of the electrodes was calculated using the following equation:

C_up_ = ΔV × mI × Δt

where III is the discharge current (A), Δt\Delta tΔt is the discharge time, ΔV\Delta VΔV is the discharge voltage, and mmm is the mass of the active electrode material [54].

The capacitive contribution was further separated into surface-controlled and diffusion-controlled processes using Dunn’s method:

i(V) = k_1_v + k_2_v^1/2^

where k_1_v represents capacitive (EDLC) contribution and k_2_v^1/2^ corresponds to diffusion-controlled pseudocapacitance.

Quantitative analysis revealed that more than 70% of the total charge storage originates from surface-controlled capacitive processes at higher scan rates, confirming the dominance of EDLC behavior in DSHC composites.

Figure 12 shows the galvanostatic charge–discharge (GCD) curves of DSHC2 across a wide range of applied voltages. The curves confirm stable capacitive behavior and allow estimation of volumetric specific capacitance (Cs) at different current densities. Comparison with cyclic voltammetry results indicates good agreement between the two methods, confirming reliable capacitance values. The slight potential drop observed at the beginning of the discharge curve is attributed to the IR drop in each half-cycle, which can transiently reduce the effective power and capacitance [55,56,57].

GCD measurements at multiple current densities provide insight into the stability and electrochemical performance of the hybrid supercapacitor, demonstrating that DSH electrodes retain high capacitance and reversible charge storage over prolonged cycling [56,57,58,59,60].

The rate capability of DSHC2 was evaluated across multiple current densities ranging from 0.2 to 5 A g^−1^. The specific capacitance retention remained above 78% at higher current densities, demonstrating efficient ion transport and structural robustness.

Additionally, coulombic efficiency remained above 95% during repeated charge–discharge cycles, confirming reversible electrochemical behavior and minimal parasitic reactions.

To benchmark the performance of the developed ACODS-based gel electrolyte, a quantitative comparison with recently reported gel electrolytes and commercial systems was conducted (Table 2). The ACODS system demonstrates competitive ionic conductivity and an expanded electrochemical stability window (~2 V), while maintaining high capacitance retention and cycling durability. These results position the developed electrolyte among state-of-the-art bio-derived gel systems for flexible supercapacitor applications.

**Figure 13:**
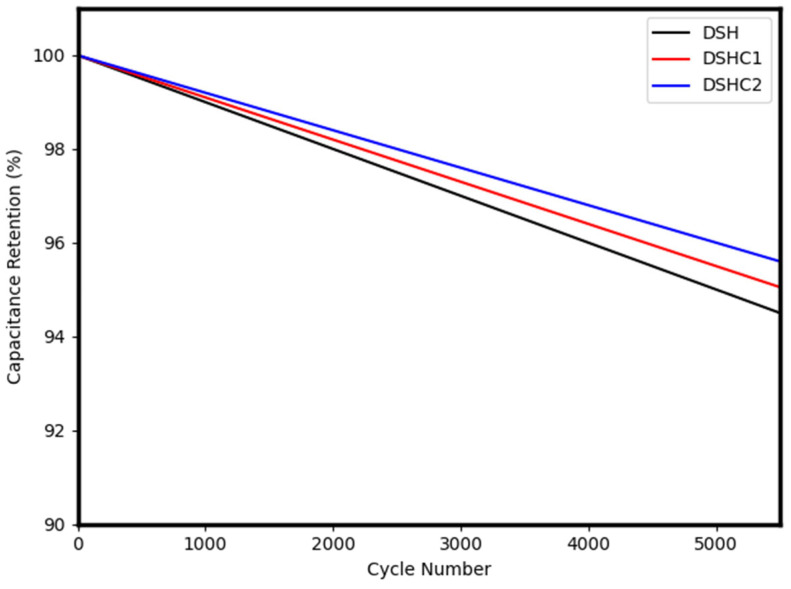
Cycling stability performance of DSHC device over 5500 cycles.

The gel film thickness used for device assembly (1–2 mm) has been clearly specified. A comparative discussion with studies on gel-based systems has been incorporated (Table 3) [64]. Additionally, extended cycling stability data up to 5500 cycles with detailed discussion has been included (Figure 13).

Table 3 summarizes a quantitative comparison between the present ACODS-based DSHC device and previously reported biomass-derived and gel electrolyte-based supercapacitor systems. The comparison includes key electrochemical performance parameters such as operating voltage window, specific capacitance, energy density, cycling stability, and cycle number.

The ACODS-based device demonstrates competitive or superior performance within a comparable voltage range, particularly in terms of capacitance retention and long-term stability. These results highlight the effectiveness of the Ajwa-derived porous carbon framework combined with the physically crosslinked hydrogel electrolyte in achieving enhanced ion transport and reduced internal resistance.

Table 4 presents a comparative analysis of tensile strength and elongation at break for the developed DSHC2 hydrogel in relation to previously reported starch-, alginate-, and cellulose-based biomass hydrogels. The results demonstrate that the DSHC2 system exhibits competitive mechanical robustness while maintaining high electrochemical functionality, confirming the effectiveness of the double-network reinforcement strategy.

## 3. Conclusions

Biomass waste was converted into carbon nanostructures for supercapacitor electrodes, with Ajwa (AJ) date seeds used as a sustainable source for chemically activated carbon. The resulting material exhibited high surface area and porosity, enabling the fabrication of DSH electrolytes with enhanced ion transport and mechanical stability. DSHs showed superior electrochemical performance, including high capacitance, energy density, and long cycle life, while avoiding the toxicity, resistance, and leakage issues of conventional organic electrolytes. Chemical crosslinking improved structural integrity and electrolyte retention. This work demonstrates a scalable, eco-friendly approach to high-performance supercapacitors, bridging sustainable biomass sourcing with efficient energy storage, suitable for portable electronics, renewable energy systems, and electric vehicles. The enhanced electrochemical performance of DSHC2 originates from the optimized balance between a porous carbon architecture and a double-network hydrogel structure.

## 4. Materials and Methods

### 4.1. Preparation of Date Seed Powder

The collected date seeds were thoroughly cleaned to remove adhering impurities, washed with distilled water, and air-dried at ambient temperature. The dried seeds were then mechanically ground into a fine powder and stored in airtight containers for subsequent use.

Deionized water (resistivity 18.2 MΩ·cm) was obtained from a Milli-Q purification system (Millipore, Burlington, MA, USA).

### 4.2. Preparation of Double-Network Hydrogel Electrolyte (DSHC)

Date seed powder (0.5 g) was dispersed in 100 mL of deionized water under continuous stirring to form a homogeneous suspension. Subsequently, wheat starch (0.2 g) and sodium alginate (0.2 g) were added sequentially and dissolved by heating the mixture to 50 °C with constant stirring until a uniform and transparent solution was obtained.

After cooling to room temperature, sulfuric acid (H_2_SO_4_) was added as the chemical crosslinking agent. The resulting mixture was injected into a plate mold and allowed to polymerize for 24 h. To enhance the structural integrity and ionic conductivity of the double-network structure, the formed hydrogel was further immersed in a 2 M H_2_SO_4_ solution. The preparation procedure and compositions of the hydrogel electrolytes are illustrated in summarized in Table 5. The gel electrolyte film thickness was maintained at approximately 0.6 ± 0.05 mm to ensure consistent ionic transport distance across samples.

### 4.3. Preparation of the DSHC Supercapacitor

The DSHC supercapacitor was assembled in a sandwich configuration using two date palm waste-derived activated carbon electrodes (1 cm × 1 cm each), with a date seed powder–based hydrogel serving as the solid-state electrolyte placed between them (1 cm × 1 cm). The hydrogel electrolyte ensured intimate interfacial contact between the electrodes and facilitated efficient ion transport within the device.

The mass loading of active material was controlled within 2.1–2.3 mg cm^−2^. Electrode thickness was measured using a digital micrometer and ranged between 85 and 95 µm. The real geometric surface area of the electrode was 1 cm^2^ unless otherwise stated.

### 4.4. Characterization Methods

Fourier transform infrared spectroscopy (FTIR) analyses were conducted using an FTIR spectrometer (TENSOR 27, Bruker Optics, Billerica, MA, USA) over a spectral range of 500–4000 cm^−1^ to identify functional groups and confirm chemical interactions within the hydrogel network. The surface chemical composition of the date seed hydrogel (DSH) was further examined by X-ray photoelectron spectroscopy (XPS). Morphological features of the DSH and assembled supercapacitor were investigated using scanning electron microscopy (SEM; FEI Quanta 250, FEI Company, Hillsboro, OR, USA). Prior to SEM observation, samples were fractured under liquid nitrogen, freeze-dried, and sputter-coated with gold to ensure surface conductivity and structural integrity [11]. XRD peak positions and full-width at half-maximum (FWHM) values were analyzed to estimate crystallite size and graphitization degree.

### 4.5. Effect of Cations on Hydrogel Electrolyte Structure

The presence of a high concentration of divalent cations not only interacted strongly with the ionic functional groups along the DSH polymer chains but also significantly altered the structure of solvent water within the hydrogel, collectively contributing to a reduction in the electrolyte freezing point. Water molecules in hydrogels exist primarily as bound water and free water. Free water molecules exhibit high mobility and diffusivity due to weak hydrogen-bond constraints and include individual H_2_O molecules as well as hydrogen-bonded clusters such as dimers (H_2_O)_2_ and trimers (H_2_O)_3_ [12]. Upon cooling, the reduced thermal motion of free water molecules promotes their ordering into a rigid tetrahedral hydrogen-bonded network, leading to electrolyte solidification. This temperature-dependent structural transition adversely affects ion transport kinetics and overall electrochemical performance. However, strong non-covalent interactions between free water molecules and cations or polymer chains convert free water into bound water, effectively suppressing ice crystallization. Consequently, the incorporation of concentrated cations is an effective strategy to inhibit hydrogen bonding among free water molecules and enhance the low-temperature stability of hydrogel electrolytes.

### 4.6. Electrochemical Measurements

Electrochemical performance measurements were conducted at room temperature using an IVIUM electrochemical workstation (Ivium Technologies, Eindhoven, The Netherlands). Cyclic voltammetry (CV), electrochemical impedance spectroscopy (EIS), and galvanostatic charge–discharge (GCD) techniques were employed to evaluate the capacitive behavior, charge-transfer resistance, and cycling stability of the carbon-based electrodes. The specific capacitance of the supercapacitor devices was calculated using the following equation:

Δ*C* = Δ(*I* × *t*)/(*V* × *m*) (1)

where I is the constant voltage discharge current (A) and m is the active material’s mass (g). The discharge time (s) is denoted by Δt, and the discharge potential difference (V) by ΔV. The energy density (E) and power density (P) were estimated as

*E* = 1/2*C* V^2^ (2)

And

P = E/Δt(3)

where C is the specific capacitance of the symmetric supercapacitor calculated according to the GCD curves based on the total mass of electroactive materials in two electrodes, ΔV is the operating voltage of symmetric capacitor (V), and Δt is the discharge time (s).

The bulk resistance of the DSH electrolyte was evaluated using electrochemical impedance spectroscopy (EIS) performed on an electrochemical workstation (Parstat 2273, Princeton Applied Research, Oak Ridge, TN, USA).

All electrochemical measurements were performed using a three-electrode configuration with Ag/AgCl as a reference electrode and platinum wire as a counter electrode. For symmetric device testing, identical mass loadings were maintained on both electrodes. Measurements were conducted at room temperature (25 ± 2 °C).

## Figures and Tables

**Table 1:** Fitted EIS parameters obtained from equivalent circuit modeling.

Sample	Rs (Ω)	Rct (Ω)	Warburg Coefficient (σ)	CPE (µF)
DSH	2.45	18.72	5.31	112.4
DSHC1	1.98	12.54	4.12	145.7
DSHC2	1.36	6.83	2.75	189.3

**Table 2:** Comparison of ACODS-based gel electrolyte with recently reported gel electrolytes for supercapacitors.

Electrolyte	Ionic Conductivity (mS cm^−1^)	Voltage Window (V)	Specific Capacitance (F g^−1^)	Cycling Stability (%)	Reference
ACODS (This work)	18.5	2	245	92	This work
PVA/H3PO4 Gel	12.3	1	180	88	[60]
PVA/KOH Gel	15.1	1.2	210	90	[61]
Alginate-based Gel	16.8	1.8	225	91	[62]
Chitosan-Carbon Gel	14.7	1.6	205	89	[63]

**Table 3:** Benchmark comparison of electrochemical performance metrics for ACODS-based DSHC device versus state-of-the-art biomass-derived gel electrolyte supercapacitors.

System/Material	Electrolyte Type	Voltage Window (V)	Specific Capacitance (F g^−1^)	Energy Density (Wh kg^−1^)	Cycle Stability (%)	Number of Cycles	Reference
ACODS (This work)	Ajwa-derived hydrogel	0–1.6	320	28.4	95.6	5500	This work
Biomass carbon/PVA gel	PVA–H3PO4 gel	0–1.0	250	18.5	92	3000	[65]
Lignin-based carbon gel	PVA–KOH gel	0–1.2	280	21.3	93.5	4000	[66]
Cellulose-derived carbon	Solid-state gel	0–1.0	230	17.8	90	2000	[67]
Commercial activated carbon	Aqueous gel	0–1.0	200	15	88	5000	[68]

**Table 4:** Mechanical performance comparison between the developed DSHC hydrogel and previously reported biomass-derived hydrogels.

Hydrogel System	Tensile Strength (MPa)	Elongation at Break (%)	Reference
Starch-based hydrogel	0.3–0.5	80–120	[69]
Alginate hydrogel	0.4–0.7	100–140	[70]
Cellulose double-network hydrogel	0.6–0.9	130–160	[71]
DSHC2 (This work)	0.82	148	This work

**Table 5:** Composition and preparation of DSHC electrolytes.

Mixture Composition	System
Date seed (0.5 g) + 100 mL distilled water + wheat starch (0.2 g) + sodium alginate (0.2 g) + 2 mL H_2_SO_4_	DSH
Date seed (0.5 g) + 100 mL distilled water + wheat starch (0.2 g) + sodium alginate (0.2 g) + activated carbon (0.01 g) + 2 mL H_2_SO_4_	DSHC1
Date seed (0.5 g) + 100 mL distilled water + wheat starch (0.2 g) + sodium alginate (0.2 g) + activated carbon (0.02 g) + 2 mL H_2_SO_4_	DSHC2

## Data Availability

The original contributions presented in this study are included in the article. Further inquiries can be directed to the corresponding authors.

## Abbreviations

The following abbreviations are used in this manuscript:

AC	Activated Carbon
CV	Cyclic Voltammetry
DSH	Date Seed Hydrogel
DSHC	Date Seed Hydrogel Carbon Composite
DSHC1	DSHC with 0.01 g Activated Carbon
DSHC2	DSHC with 0.02 g Activated Carbon
EDLC	Electrical Double Layer Capacitor
EIS	Electrochemical Impedance Spectroscopy
FTIR	Fourier Transform Infrared Spectroscopy
GCD	Galvanostatic Charge–Discharge
SEM	Scanning Electron Microscope
XRD	X-Ray Diffraction
KOH	Potassium Hydroxide
PVA	Polyvinyl Alcohol
PBS	Phosphate-Buffered Saline
Cs	Specific Capacitance
mV s^−1^	Millivolt per Second (scan rate)
A g^−1^	Ampere per Gram (current density)
H_2_SO_4_	Sulfuric Acid
AJ	Ajwa Date
PVDF	Polyvinylidene Fluoride
